# LGNN: a novel linear graph neural network algorithm

**DOI:** 10.3389/fncom.2023.1288842

**Published:** 2023-11-23

**Authors:** Shujuan Cao, Xiaoming Wang, Zhonglin Ye, Mingyuan Li, Haixing Zhao

**Affiliations:** ^1^College of Computer, Qinghai Normal University, Xining, Qinghai, China; ^2^School of Computer Science, Shaanxi Normal University, Xi’an, Shaanxi, China; ^3^The State Key Laboratory of Tibetan Intelligent Information Processing and Application, Xining, Qinghai, China; ^4^Key Laboratory of Tibetan Information Processing, Ministry of Education, Xining, Qinghai, China

**Keywords:** graph neural network, linear neural network, graph deep learning, graph representation learning, high-order structural constraint

## Abstract

The emergence of deep learning has not only brought great changes in the field of image recognition, but also achieved excellent node classification performance in graph neural networks. However, the existing graph neural network framework often uses methods based on spatial domain or spectral domain to capture network structure features. This process captures the local structural characteristics of graph data, and the convolution process has a large amount of calculation. It is necessary to use multi-channel or deep neural network structure to achieve the goal of modeling the high-order structural characteristics of the network. Therefore, this paper proposes a linear graph neural network framework [Linear Graph Neural Network (LGNN)] with superior performance. The model first preprocesses the input graph, and uses symmetric normalization and feature normalization to remove deviations in the structure and features. Then, by designing a high-order adjacency matrix propagation mechanism, LGNN enables nodes to iteratively aggregate and learn the feature information of high-order neighbors. After obtaining the node representation of the network structure, LGNN uses a simple linear mapping to maintain computational efficiency and obtain the final node representation. The experimental results show that the performance of the LGNN algorithm in some tasks is slightly worse than that of the existing mainstream graph neural network algorithms, but it shows or exceeds the machine learning performance of the existing algorithms in most graph neural network performance evaluation tasks, especially on sparse networks.

## 1 Introduction

Graph neural networks have developed rapidly in node representation learning and graph data mining in recent years. The reason why we focus on the study of graph neural networks is that data with complex network structures are common in reality, such as social networks, protein interaction networks, knowledge maps, etc. Effectively learning the representation of such graph structure data is of great significance for many tasks. The early graph neural network method is mainly based on the spectral domain or spatial domain to extract the structural information between nodes. Representative methods include graph convolutional neural network (GCN) based on spectral method (Defferrard et al., 2016) and graph sample and aggregate (GraphSage) based on spatial sampling (Hamilton et al., 2017). Both of these two methods learn node representation by aggregating node neighbor features, but there are also some limitations. Specifically, GCN relies on the calculation of the adjacency matrix of the whole graph, and it is difficult to extend to large-scale graphs; GraphSage needs to perform multiple sampling and aggregation, and the computational efficiency is low.

In order to improve the efficiency and effect of node representation learning, a variety of improvement methods are proposed in the follow-up research. The graph neural network then draws on the technologies in the field of neural networks, such as convolutional networks (Kattenborn et al., 2021), recurrent networks (Yin et al., 2017), autoencoders (Lange and Riedmiller, 2010; Liou et al., 2014; Mushtaq et al., 2022), etc., and successively proposes recursive graph neural networks (RecGNN) (Guangquan et al., 2022), convolutional graph neural networks (ConvGNN) (Duvenaud et al., 2015) and other algorithm frameworks. Although these methods extend the modeling ability of graph neural networks, they also inherit certain computational complexity. Therefore, exploring and constructing an effective graph representation learning framework has become a key goal of research.

Specifically, the linear structure is one of the simplest forms of neural networks. Convolutional networks, recurrent networks, and MLP (Pinkus, 1999) are all developed on this basis. Constructed with a simple linear framework, it not only tests the expression ability, but also ensures efficiency. Even if the performance does not exceed all existing graph neural networks, it is equivalent to or exceeds the mainstream models of existing graph neural networks in most graph neural network performance evaluation tasks, and the effectiveness of this kind of framework can be verified. For example, GRAND (Chamberlain et al., 2021) constructed a graph neural network from the perspective of differential equations for the first time, providing a principled mathematical framework. Although the results do not exceed GCN, it serves as a fulcrum to inspire follow-up research. This paper hopes to promote the research in this field by exploring the simple linear graph neural network and achieve the representation learning effect equivalent to the current model.

Among them, the method of constructing graph neural network based on simple linear structure has the following potential advantages: (1) The linear model has a simple structure, high computational efficiency, and is easier for theoretical analysis; (2) The analysis of the framework effect based on linear structure can deepen the understanding of the expression ability of graph neural network.

Under this motivation, this paper studies the construction of efficient linear graph neural networks only relying on simple linear structures without using basic neural networks such as convolution operations and activation functions, which promotes graph representation learning research and achieves comparable results with existing models.

The main contributions of this paper are as follows:

1. We propose a high-order neighbor propagation method, which can effectively capture and learn the representation information of high-order neighbor nodes without using multi-channel architecture and depth map neural network.
2. In order to capture high-quality node information, we further propose a multi-scale feature fusion mechanism, which comprehensively considers the propagation information of different orders.
3. The experimental results on multiple real data sets show that our proposed model is consistently superior to the state-of-the-art methods.

## 2 Related work

### 2.1 Shallow node vector representation

The shallow node vector representation method aims to map the nodes in the graph to low-dimensional space and learn the vector representation of node attributes and structural information. The purpose of developing shallow representation methods is to solve key challenges in graph data analysis, such as coding network topology and improving scalability. Compared with the deep model, the shallow representation has the advantages of high computational efficiency and is easier to extend to large-scale graph data. The random walk-based method generates a sequence of nodes by simulating the random walk process on the graph, and then obtains the vector representation of the nodes based on the word vector learning method. Specifically, DeepWalk (Perozzi et al., 2014) uses random walk to generate a node sequence, and then obtains a node vector representation through Word2Vec. Its innovation lies in drawing on the concept of language model in NLP and treating the node sequence as a sentence. Node2vec (Grover and Leskovec, 2016) further balances local and global structure information by adjusting the proportion of breadth-first traversal and depth-first traversal of the walk strategy, that is, Node2vec can control whether the walk is more in accordance with the network neighbor expansion or more choices to re-hop. LINE (Tang et al., 2015) preserves the first-order node co-occurrence relationship and second-order node similarity by constructing first-order and second-order prox word vectors. These methods generally encode network structure information by walking. In summary, the shallow node vector representation method provides a simple and effective node representation learning method. The above methods are all devoted to encoding the information in the network topology and providing information-rich node vector representations for various graph analysis tasks.

### 2.2 Graph neural network

Graph neural network is an important technical direction in graph representation learning and analysis in recent years. It shows amazing modeling ability and expression ability in various graph learning tasks. The key idea is to develop a deep learning model that can effectively process graph structure data by referring to neural networks. For example, the Chebyshev graph convolutional neural network ChebyNet (Defferrard et al., 2016) is an early model for fusing graph convolution operations, where the key is the Chebyshev polynomial approximation graph convolution operation. ChebyNet pioneered the application of convolutional network ideas on graph data. GCN further proposes a method of convolution directly in the graph field, and performs feature aggregation through the Laplacian matrix. The introduction of GCN has promoted the wide application of direct graph convolution in various tasks. Based on GCN, GraphSAGE generates node representation by layer-by-layer aggregation of neighbor embedding, which has high scalability and generalization ability and can be extended to large-scale graphs. The graph attention network (GAT) (Veličković et al., 2018) introduces an attention mechanism, which allows different nodes to assign different weights to neighbors, enhances the model ‘s ability to capture local information of graph structure, brings significant performance improvement for different application scenarios, and promotes the development of this research field. Simplify graph convolutional network.

Graph neural networks are used to learn the node representation of graph-structured data, while the current mainstream technology frameworks can be divided into two categories: graph convolution-based frameworks and graph sampling-based frameworks (Wu et al., 2019).

Table 1 shows the differences among GCN, GraphSage and Linear Graph Neural Network (LGNN). We select several capabilities as different perspectives for comparison: whether high-order neighborhood information is learned during training (Higher-order information), whether activation function is used for transformation (Activation function), whether the information transmitted by multi-order neighbors is aggregated (Multi-order neighbor propagation), whether the degree of influence of different order domain information is considered (Different order neighborhood influence), and whether the message passing mechanism is designed (Message passing mechanism).

**Table 1:** Differences of GCN, GraphSage and LGNN.

Model	Higher-order information	Activation function	Multi-order neighbor propagation	Different order neighborhood influence	Message passing mechanism
GCN	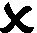	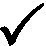	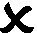	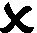	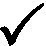
GraphSage	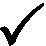	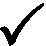	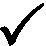	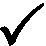	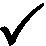
LGNN	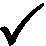	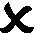	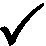	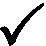	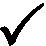

## 3 Methodology

**Figure 1:**
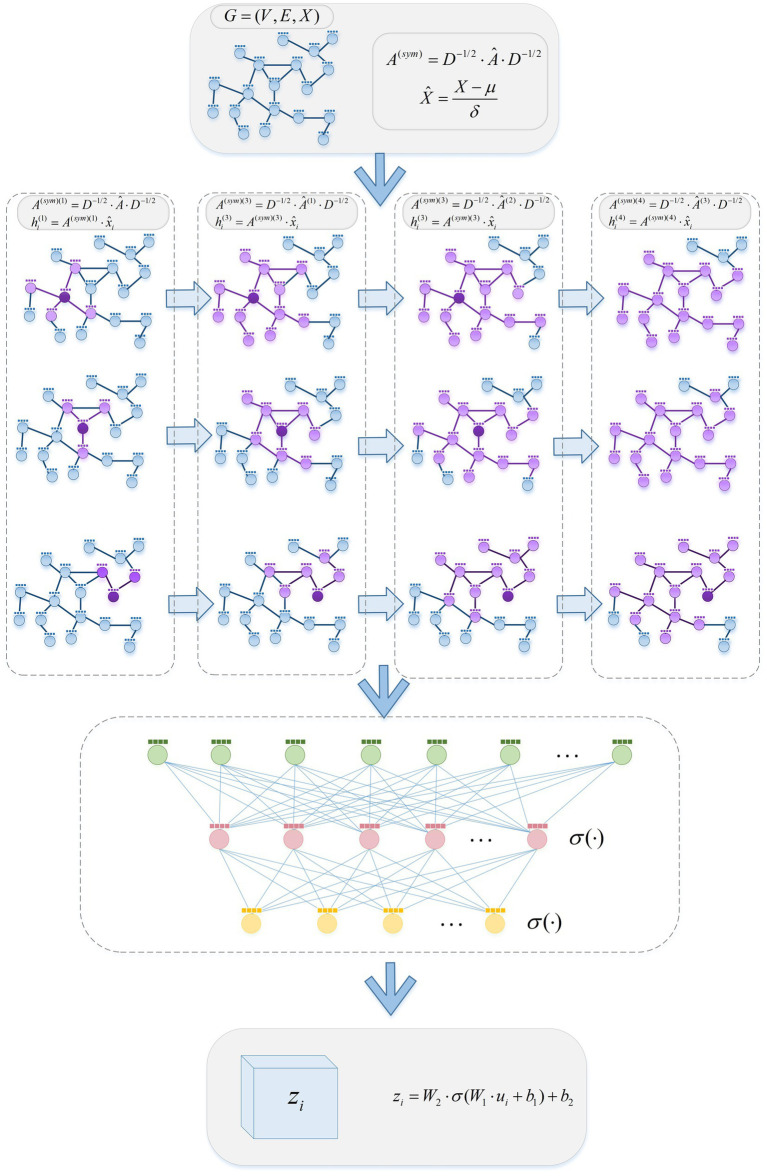
LGNN network structure.

Aiming at the problem of insufficient perception of high-order neighborhood information in shallow linear networks, this paper proposes a LGNN neural network, and its structure is set as shown in Figure 1.

Firstly, in the graph representation and preprocessing stage, the input graph is subjected to symmetric normalization and feature normalization preprocessing to obtain a normalized adjacency matrix and a feature matrix. Secondly, by designing a high-order adjacency matrix propagation mechanism, the high-order neighbor feature information of the node is iteratively aggregated, and the final node representation information is obtained through efficient linear transformation mapping.

### 3.1 Graph representation and preprocessing

Considering an undirected graph G=(V,E,X) , there are nodes $v_{i}\in V$ and edges $\left(V_{i},V_{j}\right)\in E$ . In order to solve the problem that a single-channel shallow graph neural network cannot capture high-order structures, LGNN uses the same depth of GCN and GraphSage, that is, a two-layer fully connected neural network structure. In addition, no longer use any methods and components commonly used in neural network structures, such as convolution operations and activation functions.

For each node $v_{i}\in V$ , there is an initial feature representation $x_{i}\in R^{d}$ , where d is the dimension of the feature. In the LGNN model, we use the adjacency matrix $A\in R^{N\times N}$ to represent the topology of the graph, where $A_{ij}=1$ denotes that there are edges between nodes $v_{i}$ and $v_{j}$ , and $A_{ij}=0$ denotes that there are no edges between them. In order to prevent the loss of information, we symmetrically normalize the adjacency matrix:


(1) $$A^{\left(sym\right)}=D^{-1/2}\cdot \hat{A}\cdot D^{-1/2}$$

Among them, $\hat{A}=A+I$ represents the self-ring matrix added on the basis of the adjacency matrix, D is the degree matrix, $D_{ii}=\underset{i=0}{\sum }{\hat{A}}_{ij}$ . Considering the deviation of node features, removing the influence of node degree and obtaining more uniform representation, we define it as:


(2) $$\hat{X}=\frac{X-\mu }{\delta }$$

Among them, $\hat{X}$ represents the normalized feature matrix, μ and δ are the mean and standard deviation of the feature matrix X , respectively. Through the standardized method, different node features have similar scales, which improves the convergence speed and performance of the LGNN model.

### 3.2 Higher-order neighbor propagation

In order to capture the complex dependencies between nodes in the graph more deeply, we propose a feature learning method based on high-order adjacency matrix propagation. Based on the given node feature matrix $\hat{X}$ and normalized $A^{\left(sym\right)}$ , we design a linear propagation method to explore the association between nodes at different orders. In *k*-order propagation, we calculate the update equation of node characteristics as follows:


(3) $$h_{i}^{\left(k\right)}=A^{\left(sym\right)}\cdot {\hat{x}}_{i}$$

(4) $$A^{\left(sym\right)\left(k\right)}=\overset{k}{\underset{i=1}{\prod }}A^{\left(sym\right)\left(i\right)}$$

Here, $h_{i}^{\left(k\right)}$ represents the *k*-th-order post-propagation node feature representation. Through this way of communication, the node features can capture high-order neighbor information, so as to better reflect the characteristics of nodes in high-order hierarchical relationships. In order to comprehensively utilize the propagation information of different orders, we introduce a multi-scale feature fusion mechanism. Specifically, we multiply the node features of each order by a corresponding weight matrix, and then stack the features of all orders according to the channel direction to form a multi-scale node feature tensor:


(5) $$z_{i}=msf\left(\left[h_{i}^{\left(1\right)}\cdot W^{\left(1\right)}∥h_{i}^{\left(2\right)}\cdot W^{\left(2\right)}\right]∥\ldots ∥h_{i}^{\left(k\right)}\cdot W^{\left(k\right)}\right)$$

Among them, msf(·) represents the multi-scale fusion method, $W^{\left(1\right)},\ldots ,W^{\left(k\right)}$ represents the weight matrix of the corresponding order during propagation, and || represents the tensor stitching method. This multi-scale feature fusion mechanism allows LGNN to extract rich information from the propagation of different orders, thus improving the understanding and expression ability of graph structure. In order to avoid the problem of gradient disappearance during the propagation of multi-order adjacency matrix, we introduce a normalization method to maintain the stable propagation of gradient:


(6) $$u_{i}=\frac{{\hat{x}}_{i}-\frac{1}{m}\sum_{j=1}^{m}{\hat{x}}_{i}}{\sqrt{\frac{1}{m}\sum_{j=1}^{m}{\left({\hat{x}}_{i}-\frac{1}{m}\sum_{j=1}^{m}{\hat{x}}_{i}\right)}^{2}+\xi }}$$

Among them, ξ is a constant to avoid divisor zero. In addition, we also use multi-layer perceptron (MLP) to map features to the semantic space to further enhance the expression ability of features:


(7) $$z_{i}=W_{2}\cdot \sigma \left(W_{1}\cdot u_{i}+b_{1}\right)+b_{2}$$

### 3.3 Training and optimization

The loss function for model training consists of two parts, the first part is the negative log-likelihood loss function, whose mathematical expression:


(8) Loss=l1+l2

(9) $$l1=-\frac{1}{N}\overset{N}{\underset{i=1}{\sum }}\log\left(softmax\left(lgits_{i}\right)\left[labels_{i}\right]\right)$$

Where $lgits_{i}$ is the output of the model, a tensor containing the log probability values of the predicted outcomes, and $labels_{i}$ is the true labeled values. The function softmax(·) converts the log probability values to probability values. This loss function calculates the difference between the model prediction and the true label by comparing the predicted probability distribution with the true label. The second part *l*2 is the L2 regularization term, which is added by adding a penalty term after the loss function. The purpose of this is to minimize the negative log-likelihood loss while constraining the weights of the model through the regularization term in order to improve the generalization ability of the model and prevent overfitting.

That is, the objective function of this paper is described as follows:

Let the training dataset be: $D=\left(x_{1},y_{1}\right),\left(x_{2},y_{2}\right),\ldots ,\left(x_{n},y_{n}\right)$ , where: $x_{i}\in {\mathbb{R}}^{d}$ represents the d-dimensional feature vector of the *i*-th training sample. $y_{i}\in \left\{1,2,\ldots ,k\right\}$ represents the label of the *i-th* training sample, with a total of k categories. The model parameters are denoted as θ . The model’s prediction probability for category $y_{i}$ of sample $x_{i}$ is: $P_{model}\left(y_{i}|x_{i};\theta \right)$ .

The loss function is the negative log-likelihood loss function:


(10) $$L\left(\theta \right)=-\sum_{i=1}^{n}\log\left(P_{model}\left(y_{i}|x_{i};\theta \right)\right)$$

The loss function after adding the L2 regularization term is:


(11) $$L_{reg}\left(\theta \right)=L\left(\theta \right)+\frac{\lambda }{2}{\left\|\theta \right\|}_{2}^{2}$$

where λ is the regularization coefficient, ${\left\|\theta \right\|}_{2}^{2}=\sum_{j=1}^{D}\theta_{j}^{2},$ $\theta_{j}$ is the *j*-th element of the model parameter θ , and *d* is the dimension of the parameter θ .

Classical graph neural network approaches such as Graph Convolutional Network (GCN) and GraphSage achieve superior performance not only because of the powerful neural network architectures employed, but more importantly, because of the ability of these models to efficiently encode the structural information and feature representations of the input graph. In fact, recent findings have shown that reasonably effective structural feature engineering is no less important to the graph learning task than the expressive power of the model itself (Dwivedi and Bresson, 2020).

LGNN is a lightweight, linear graph filtering layer based on the Laplace matrix of graphs. Through theoretical analysis, we demonstrate that a simple linear operation can effectively aggregate the structural information of nodes. In addition, we design a multi-layer superimposed network structure to enhance the representation capability of the model. LGNN provides a new idea for GNN model design, i.e., mechanisms with lower computational and spatial complexity should be prioritized while maintaining the representation capability.

## 4 Experiments and discussion

### 4.1 Experimental datasets

The experiment used three literature citation network datasets of Cora, Citeseer and PubMed, and we published the datasets on GitHub*. All three are an undirected graph with nodes representing documents (thesis documents) and edges representing citation relationships. Table 2 summarizes the statistics of the three benchmark datasets - Cora, Citeseer, and PubMed. We used exactly the same experimental setup on these three benchmark datasets as in the semi-supervised graph mining literature. Such as feature and data segmentation, and ran 100 trials with 100 random seeds for all results on Cora, Citeseer, and PubMed reported in Section 4.

**Table 2:** Statistical data for the three benchmark datasets.

Datasets	Node	Edge	Training/effective/test nodes	Categories	Feature
Cora	2,708	5,429	140/500/1,000	7	1,433
CiteSeer	3,327	4,732	120/500/1,000	6	3,703
PubMed	19,717	44,338	60/500/1,000	3	500

### 4.2 Experimental setup

The experiment uses the PyTorch framework to implement the LGNN model and the entire training and testing process. The experimental environment is Windows operating system, and the PyTorch version is 11.7.

The evaluation indexes used in the experiment are accuracy, recall, and F1 value, the specific formula is:


(12) $$Acc=\frac{N_{pre\_right}}{N_{pre}},$$

(13) $$F_{1}=\frac{2\times Acc\times Recall}{Acc+Recall},$$

Among them, $N_{pre\_right}$ is the number of correct LGNN predictions in the test sample, and $N_{pre}$ is the total number of test samples.

All the results on Cora, Citeseer and PubMed reported in Section 4.1 of this paper were run 100 trials with 100 random seeds.

For all data sets, we do not use the dropout operation, and use the Adam optimizer to set the weight attenuation coefficient and the L2 regularization coefficient. The number of neurons in the hidden layer represents the vector length, and the optimizer learning rate is shown in Table 3.

**Table 3:** Detailed parameter setting.

Dataset	Training epochs	Learning rate	Weight decay	Hidden dimension	Activation function
Cora	100	0.01	5e-3	128	Relu
Citeseer	100	0.01	5e-4	256	Prelu
PubMed	500	0.01	5e-3	512	Relu

### 4.3 Comparison algorithms

In this paper, two kinds of comparative experiments are set up. The first kind is the traditional network representation learning method, such as Node2Vec and DeepWalk. The second kind is the graph neural network method, such as GCN, GAT, GraphSage, APPNP, Graph U-Net.

**Figure 2:**
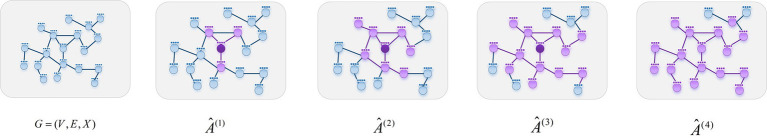
State diagram of different propagation orders.

Take any node in the graph *G*, set the state of the k={1,2,3,4} -order propagation as shown in Figure 2, and the node characteristics after propagation are represented as follows:


(14) $$\left\{ \begin{array}{l} h_{i}^{\left(1\right)}=A^{\left(sym\right)\left(1\right)}\cdot {\hat{x}}_{i}\\ h_{i}^{\left(2\right)}=A^{\left(sym\right)\left(2\right)}\cdot {\hat{x}}_{i}\\ h_{i}^{\left(3\right)}=A^{\left(sym\right)\left(3\right)}\cdot {\hat{x}}_{i}\\ h_{i}^{\left(4\right)}=A^{\left(sym\right)\left(4\right)}\cdot {\hat{x}}_{i} \end{array}\right.$$

In this paper, several variations are proposed for the proposed LGNNs, which are described as follows:

*LGNNoriginal*: in each input feature, the representation vector of the node is not multiplied with $\hat{A}$ . The features of the node are directly input, and then a two-layer fully connected network is built for training.

*LGNN1*: in each input feature, the feature matrix of the node is multiplied with ${\hat{A}}^{\left(1\right)}$ , and then a two-layer fully connected network is built for training.

*LGNN2*: in each input feature, the feature matrix of the node is multiplied with ${\hat{A}}^{\left(1\right)}$ and ${\hat{A}}^{\left(2\right)}$ respectively, and the result is two feature matrices, after which the two feature matrices are spliced horizontally, and each row of which is used as an input feature in the algorithm of this paper, and then a two-layered fully-connected network is constructed for training.

*LGNN3*: in each input feature, the feature matrix of the node is multiplied with ${\hat{A}}^{\left(1\right)}$ , ${\hat{A}}^{\left(2\right)}$ , ${\hat{A}}^{\left(3\right)}$ , and the result is three feature matrices, after which these three feature matrices are spliced horizontally, and each row of which is used as an input feature in the algorithm of this paper. Then a two-layer fully connected network is constructed for training.

*LGNN4*: each time the input features, the node’s feature matrix is multiplied with ${\hat{A}}^{\left(1\right)}$ , ${\hat{A}}^{\left(2\right)}$ , ${\hat{A}}^{\left(3\right)}$ and ${\hat{A}}^{\left(4\right)}$ , the result is five feature matrices, and then these five feature matrices are spliced horizontally, and each row is used as an input feature in the algorithm of this paper, and then a two-layered fully-connected network is constructed for training.

### 4.4 Experimental results and analysis

The experiment tests the performance of the model on the public data set and verifies the performance and flexibility of the model. Table 4 shows the accuracy indicators of various baseline neural networks and LGNNs in Cora, Citeseer, and PubMed datasets when performing downstream tasks for node classification.

**Table 4:** Comparison of node classification accuracies.

Methods	Cora	CiteSeer	PubMed
GCN	81.40	70.3	79.0
Node2Vec	74.8	52.3	80.3
DeepWalk	75.7	50.5	80.5
SGC	81.60	69.1	84.8
GAT	80.20 ± 0.7	72.5 ± 0.7	79.0 ± 0.3
APPNP	83.8 ± 0.3	71.6 ± 0.5	79.7 ± 0.3
Graph U-Net	78.9 ± 1.0	67.4 ± 0.7	77.8 ± 0.6
LGNNoriginal	66.03 ± 1.51%	62.13 ± 1.54%	66.56 ± 2.07%
LGNN_1_	79.53 ± 1.44%	74.56 ± 1.97%	72.01 ± 2.09%
LGNN_2_	81.37 ± 1.31%	**75.64 ± 2.13%**	75.74 ± 1.90%
LGNN_3_	81.47 ± 1.15%	75.54 ± 1.97%	**76.23 ± 1.84%**
LGNN_4_	**82.50 ± 1.16%**	74.92 ± 1.41%	OOM

Bold value means best performed method.

From the Table 4, it can be seen that on the Cora dataset, LGNN4 performs better, reaching 82.50% accuracy, which is at the level of runner-up; on the Citeseer dataset, LGNN2 reaches the level of champion compared with other algorithms, increasing by 3.14% ~ 25.34%; on the PubMed dataset, LGNN3 is slightly lower than some comparison algorithms, but it is still competitive compared to other algorithms. We can get the following conclusions:

1. The multi-order adjacency matrix propagation of LGNN enables it to iteratively transmit information in the graph structure, so as to better capture the complex relationship between nodes. Multi-order adjacency matrix propagation enables LGNN to flexibly adapt to the topology of different graphs and improves the generalization of LGNN.
2. The connection between nodes in the Citeseer dataset is sparse, and the path of information transmission is relatively limited. Compared with other comparison models, LGNN transmits information on a limited path with a multi-order adjacency matrix, and uses multi-scale high-order feature fusion to further avoid the problem of excessive accumulation of information transmission in dense graphs.
3. The multi-scale feature fusion mechanism of LGNN allows the model to make full use of the features obtained by different order propagation and map them to a shared semantic space, so that the model can understand the semantic information in the graph more comprehensively. This feature fusion ability enables LGNN to classify nodes more accurately. The reason why it performs well on the Citeseer dataset is that the connection between nodes on the sparse dataset is sparse, and the path of information transmission is relatively limited. Compared with other comparison models, LGNN transmits information on a limited path with a multi-order adjacency matrix, and uses multi-scale high-order feature fusion to further avoid the problem of excessive accumulation of information transmission in dense graphs.

The Micro-F1 value indicators of LGNN on Cora, Citeseer and PubMed datasets are shown in Table 5. Observing these results, it can be found that different versions of LGNN show their own advantages in node classification tasks on different data sets.

1. On the Cora dataset, as LGNN is gradually improved from version 1 (LGNN1) to version 4 (LGNN4), the Micro-F1 value shows a gradual upward trend. The reason behind this improvement can be attributed to the increasing complexity of the LGNN model. With the iteration of the version, LGNN introduces deeper layers, higher-order adjacency matrix propagation and more complex feature fusion mechanism, which makes the model better capture the features of nodes in the Cora dataset. Finally, the Micro-F1 value of 82.5% is achieved in the LGNN4 version.
2. On the Citeseer dataset, LGNN2 performs well, and its performance is significantly improved compared to the initial version (LGNN1). This improvement is mainly due to the introduction of higher-order adjacency matrix propagation and finer feature fusion mechanism in LGNN2. This improvement enables LGNN2 to handle Citeseer better.
3. On the PubMed dataset, LGNN3 performs better than other versions, especially when dealing with large-scale and sparse graph data. This may be because the model design and feature learning ability of LGNN3 are stronger, so that it can better adapt to the characteristics of PubMed data and improve the accuracy of node classification.

**Table 5:** Comparison of node classification Micro-F1 values.

Methods	Cora	CiteSeer	PubMed
LGNN_original_	0.6603 ± 0.0139	0.6213 ± 0.0151	0.6656 ± 0.0207
LGNN_1_	0.7953 ± 0.0144	0.7456 ± 0.0177	0.7201 ± 0.0213
LGNN_2_	0.8137 ± 0.0128	**0.7564 ± 0.0204**	0.7574 ± 0.0190
LGNN_3_	0.8147 ± 0.0115	0.7554 ± 0.0190	**0.7623 ± 0.0184**
LGNN_5_	**0.8250 ± 0.0116**	0.7492 ± 0.0131	OOM

Bold value means best performed method.

### 4.5 Parameter sensitivity

**Figure 3:**
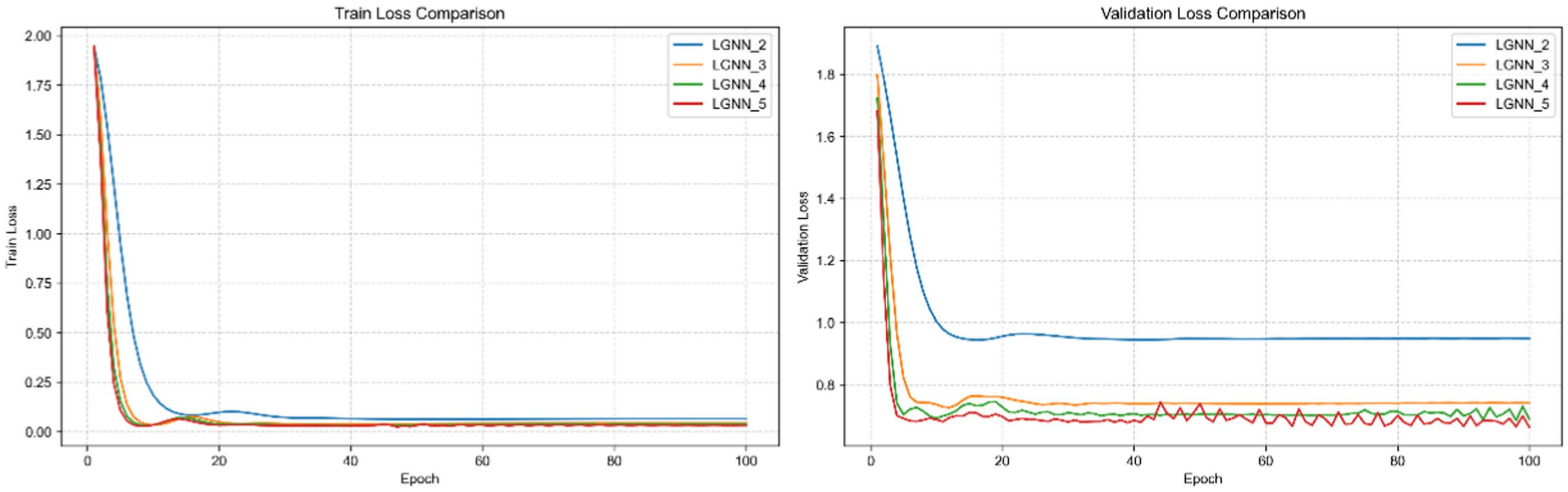
Training loss and verification loss between different algorithms on Cora dataset.

**Figure 4:**
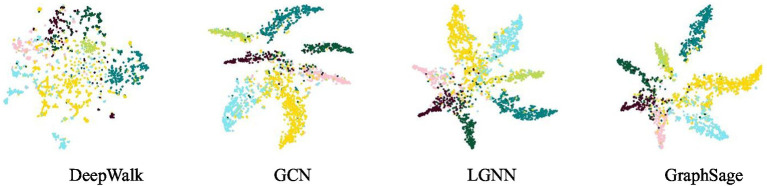
Visualization of t-SNE represented by nodes on Cora. From left to right are the visualization results of DeepWalk, GCN, LGNN and GraphSage.

In Figure 3, the training loss and verification loss of four variants of LGNN, LGNN1, LGNN2, LGNN3 and LGNN4, on the Cora dataset are compared. Compared with LGNN1, LGNN2 has more feature fusion of second-order neighbors, so its performance is improved. LGNN3 and LGNN4 have successively added higher-order structural information, so the loss rate continues to decline. When the power of the adjacency matrix increases, the structural information that the nodes can aggregate is richer, so the model’s ability to model the network topology is enhanced, and the performance is improved.

t-SNE is a machine learning algorithm for data dimensionality reduction, which can capture the local structure and global structure in high-dimensional data and reflect the discriminative ability of node representation in the graph (Van Der Maaten and Hinton, 2008). We randomly select nodes from Cora and use t-SNE to map their embeddings to a two-dimensional space. These embedded visualizations are shown in Figure 4. It can be seen from the graph that the embedding distribution of DeepWalk shows an excessively uniform distribution in the embedding space, indicating that there is no clear graph structure to capture deep information well. Compared with DeepWalk, LGNN has made great progress, which indicates that the message passing mechanism is conducive to the model learning discriminative node representation. Compared with GCN and GraphSage, our method can still identify clear structures to capture the collaboration effect to the same extent without using graph convolution and activation function, and the embedding in each class is reasonably dispersed to reflect the feature information of the graph.

We use different dropout ratios (0.1–0.5) on the two graph node classification data sets of Cora and Citeseer, and conduct experiments on the neural network based on the LGNN4 algorithm to compare and analyze the impact of different dropout operations on node classification.

The results are shown in Table 6. We observe that on the Cora dataset, with the increase of dropout ratio, the classification accuracy increases slightly from 82.44 to 83.33%. On the Citeseer data, the classification accuracy varies from 75.57 to 76.10%. On the two data sets investigated, the following conclusions can be drawn:

1. On the Cora dataset, the highest accuracy of 83.33% is obtained when the dropout ratio is 0.3, while on the Citeseer dataset, the dropout ratio of 0.3 makes the accuracy reach the highest point of 76.10%. This shows that a moderate dropout operation helps to improve the robustness of LGNN, but an excessive dropout ratio may reduce the classification performance of LGNN.
2. Under the same dropout ratio, the accuracy of the Cora dataset is generally higher than that of the Citeseer dataset. This may be because the Cora data set is relatively small and the relationship between nodes is more intensive, while the Citeseer data set is larger and the relationship between nodes is sparser. Therefore, for dense graph data, dropout operation can better improve the robustness of LGNN, thus improving the accuracy of node classification.
3. It can be seen from the standard deviation of the results that as the dropout ratio increases, the performance volatility of the model also increases. This indicates that a higher dropout ratio may introduce instability, which makes the performance of the model vary greatly in different training iterations. Therefore, when choosing the dropout ratio, it is necessary to choose an appropriate value to synthesize LGNN performance and stability.

**Table 6:** Analysis of the effect of dropout on node classification on Cora and CiteSeer datasets.

Dropout	Cora	CiteSeer
0.1	82.44 ± 0.70%	75.57 ± 1.15%
0.2	82.79 ± 0.75%	75.93 ± 0.75%
0.3	83.33 ± 0.96%	76.10 ± 0.69%
0.4	83.25 ± 0.75%	76.03 ± 0.56%
0.5	82.90 ± 1.25%	76.00 ± 0.66%

## 5 Conclusion

Most of the current graph neural networks use traditional neural network components (such as convolution operations and activation functions) to capture the characteristics of neighbors. Therefore, we propose a simple and effective feature learning method LGNN for high-order adjacency matrix propagation. Through efficient and concise linear operations based only on graph Laplacian matrices, it is sufficient for graph neural networks to learn high-quality node representations without complex nonlinear convolution or aggregation operations. Experiments show that our method can achieve or exceed the effect of existing baselines on most data sets, especially for sparse data sets. In the future, we will further study and extend LGNN to large-scale data sets and focus on how to effectively model structures and extract effective structural information, rather than simply pursuing model complexity.

## Data availability statement

The original contributions presented in the study are included in the article/supplementary material, further inquiries can be directed to the corresponding author.

## Author contributions

SC: Conceptualization, Methodology, Software, Writing – original draft. XW: Supervision, Writing – review & editing. ZY: Funding acquisition, Supervision, Writing – review & editing. ML: Writing – review & editing. HZ: Supervision, Writing – review & editing.
